# Clearance of Type-Specific, Low-Risk, and High-Risk Cervical Human Papillomavirus Infections in HIV-Negative and HIV-Positive Women

**DOI:** 10.1200/JGO.17.00129

**Published:** 2018-08-20

**Authors:** Sally N. Adebamowo, Ayotunde Famooto, Eileen O. Dareng, Oluwatoyosi Olawande, Olayinka Olaniyan, Richard Offiong, Clement A. Adebamowo, for the ACCME Research Group as part of the H3Africa Consortium

**Affiliations:** **Sally N. Adebamowo** and **Clement A. Adebamowo**, University of Maryland School of Medicine, Baltimore, MD; **Sally N. Adebamowo**, **Ayotunde Famooto**, and **Clement A. Adebamowo**, Center for Bioethics and Research, Ibadan; **Ayotunde Famooto** and **Oluwatoyosi Olawande**, Institute of Human Virology Nigeria; **Olayinka Olaniyan**, National Hospital Abuja; **Richard Offiong**, University of Abuja Teaching Hospital, Abuja, Nigeria; **Eileen O. Dareng**, University of Cambridge, Cambridge, United Kingdom.

## Abstract

**Purpose:**

There is a dearth of data on clearance of cervical human papillomavirus (HPV) infection among women in West Africa. We examined the clearance of low-risk (lr) and high-risk (hr) cervical HPV infections, and the factors associated with these measures in HIV-negative and HIV-positive women.

**Methods:**

We studied 630 Nigerian women involved in a study of HPV infection using short polymerase chain reaction fragment-10 assay and line probe assay-25. Research nurses used a cervical brush to collect samples of exfoliated cervical cells from all the study participants. Cox proportional hazards models were used to estimate associations between HIV and HPV infections.

**Results:**

The mean age of the study participants was 38 (standard deviation, ± 8) years; 51% were HIV positive. The rate of clearing any HPV infection was 2.0% per month among all women in the study population, 2.5% per month among HIV-negative women, and 1.6% per month, among HIV-positive women. The clearance rate per 1,000 person-months of observation for any lrHPV infection and any hrHPV infection were 9.21 and 8.83, respectively, for HIV-negative women, and 9.38 and 9.37, respectively, for HIV-positive women. In multivariate models, the hazard ratios for HIV-positive compared with HIV-negative women were 0.85 (95% CI, 0.51 to 1.43; *P* = .55) and 0.95 (95% CI, 0.54 to 1.65; *P* = .85) for cleared infections with any lrHPV and any hrHPV, respectively. The hazard ratio for HIV-positive compared with HIV-negative women was 0.39 (95% CI, 0.17 to 0.88; *P* = .02) for cleared infections with any multiple HPV and 0.13 (95% CI, 0.03 to 0.58; *P* = .007) for cleared infections with multiple hrHPV.

**Conclusion:**

In this study population, we observed that HIV-positive women were less likely to clear infections with multiple hrHPV types.

## INTRODUCTION

Human papillomaviruses (HPVs) are highly prevalent infections in most of the world. They primarily infect stratified cutaneous or mucosal epithelia.^1^ Mucosotropic HPVs are subdivided into low-risk HPV (lrHPV) and high-risk HPV (hrHPV), depending on their association with human cancers.^1^ The lrHPVs are mainly associated with anogenital warts; hrHPVs are associated with anogenital cancers, including cervical cancer. Globally, HPV-16 and -18 contribute to > 70% of all cervical cancer cases; after HPV-16 and -18, the six most common HPV types are HPV-31, -33, -35, -45, -52, and -58, and they account for an additional 20% of cervical cancers worldwide.^2^

Approximately 70% of HPV infections are cleared spontaneously in 1 year and 90% in 2 years; the infection persists in the remainder of cases.^3^ Although persistent infection with hrHPV genotypes is not sufficient to cause cancer, it increases the risk of progression to cancer. HPV clearance requires an effective cell-mediated immune response.^4^ Therefore, HIV-positive individuals who become infected with HPV are less likely to clear the infections within 1 to 2 years and thus have increased risk of developing benign warts and malignant tumors. Given the dearth of data from sub-Saharan Africa, the objective of this study was to examine the clearance rates of type-specific low-risk and high-risk cervical HPV infections, and identify the associated risk factors among Nigerian women.

## MATERIALS AND METHODS

### Study Population

The study participants were 1,020 women enrolled from cervical cancer clinics at National Hospital Abuja and University of Abuja Teaching Hospital, Nigeria, between 2012 and 2014. All the study participants were age ≥ 18 years, had history of prior vaginal sex, were not pregnant, and had an intact uterus at enrollment. Interviewers used questionnaires to collect data on sociodemographic characteristics, sexual and reproductive history, and HIV status, which were confirmed from medical records. Research nurses collected blood samples from the antecubital veins of and performed pelvic examinations on all the study participants at enrollment and at the follow-up visit, which was scheduled to occur 6 months after enrollment. In this analysis, we included 630 women who completed the study procedures at the baseline and follow-up visits.

### HPV Detection by SPF_10_/LiPA_25_

We extracted DNA from cervical exfoliated cells, as previously described.^5^ Samples were tested for the presence of HPV DNA by hybridization of short polymerase chain reaction fragment-10 (SPF_10_) amplimers to a mixture of general HPV probes recognizing a broad range of high-risk, low-risk, and possible hrHPV genotypes in a microtiter plate format, as described previously.^6^ All samples determined to be HPV DNA positive by SPF_10_ DNA enzyme immunoassay were genotyped using line probe assay-25 (LiPA_25_). The LiPA_25_ assay provides type-specific information for 25 different HPV genotypes simultaneously and identifies infection by one or more of 13 hrHPV genotypes: 16, 18, 31, 33, 35, 39, 45, 51, 52, 56, 58, 59, and 68.^7,8^ However, the test cannot differentiate between HPV 68 and 73, so we defined this HPV genotype, HPV-68/73, as low risk. The test can identify lrHPV types 6, 11, 34, 40, 42, 43, 44, 53, 54, 66, 70, and 74. The test also identifies unspecified HPV genotypes, which we defined as low risk. We defined HPV infection as prevalent if at least one HPV genotype was detected by the HPV test (SPF_10_/LiPA_25_) in a sample provided at the baseline visit and as cleared if the prevalent HPV genotype was not detected during the follow-up visit.

### Statistical Analysis

A study conducted among Senegalese women showed that the hazard ratio (HR) of HPV clearance among HIV-positive women was 0.31, compared with HIV-negative women.^9^ Using G*Power statistical software (http://www.gpower.hhu.de),^10,11^ we computed the minimum sample size required to detect a 31% difference based on *t* test for difference between two independent means (two-sided tests with α = 0.05 and β = 0.8 [ie, 80% power]) as 165 per group.^10,11^ Our sample size of 309 HIV-negative and 321 HIV-positive women provided 90% power, which was adequate to detect a difference of about 25% between the groups.

To compute socioeconomic status (SES) in a low-resource environment where income data are sparse, we generated wealth index data, as previously described.^12^ In summary, we used principal components analysis with varimax rotation to compute factor scores based on the sum of the ownership of household items weighted by their factor loading. We sorted the data on the first principal component, which had the highest eigenvalue, and divided all respondents into three categories based on its value. Participants in the lowest 40% were categorized as low SES, the middle 40% were categorized as middle SES, and the top 20% were categorized as high SES. The validity and reproducibility of the wealth index have been examined in previous studies and it correlates well with other measures of wealth in environments without reliable expenditure data.^12^

We used *t* tests to assess differences in the distribution of continuous variables between groups, and χ^2^ and Fisher exact tests for categorical variables. Person-months of observation (PMO) were calculated from the date of the baseline visit to the date of follow-up visit. We used Kaplan-Meier curves and log-rank test of homogeneity to examine the clearance of HPV infections over time. To examine associations between potential risk factors and cleared lrHPV, and hrHPV infections, we used Cox proportional hazards models stratified by age to estimate HRs and 95% CIs. Our main analyses compared the impact of HIV infection on clearance of lrHPV and hrHPV. HRs < 1.0, therefore, are consistent with lower clearance rates (more persistent infections) for the category being evaluated relative to the referent category. For the multivariate analysis, we used stepwise-regression models with *P* < .20 as the entry criterion and *P* < .10 as the threshold to stay in the model. Age and HIV were kept in the model throughout the selection process. We selected those variables that were associated with HPV at *P* < .20 for inclusion in the multivariate models. All the reported *P* values were two sided. All the analyses were performed using SAS, version 9.3, for UNIX statistical software (SAS Institute, Cary, NC).

### Ethics

The study was conducted according to the Nigerian National Code for Health Research Ethics. Ethical approval to conduct this study was obtained from the Nigerian health research ethics committee. Informed consent was obtained from all participants before enrollment in the study.

## RESULTS

Of the 1,020 participants enrolled at baseline, 71% (725 of 1,020) attended the follow-up visit. We excluded 95 participants because of missing results (n = 20 missing HIV results; n = 9 missing baseline visit HPV results; n = 59 missing follow-up visit HPV results; n = 4 missing HIV and baseline HPV results; n = 1 missing HIV and follow-up visit HPV results; and n = 2 missing HPV results at the baseline and follow-up visits) and included the remaining 630 women (62%) in the analysis for HPV clearance. The follow-up visit occurred at a median time of 9 months after the baseline visit.

The mean age of the study participants was 38 (standard deviation, ± 8) years and about half (49%, 309 of 630) were HIV negative (Table 1). At baseline, 41% (261 of 630) of the study participants tested positive for infections with any type of HPV, 25% (160 of 630) tested positive for lrHPV and 25% (155 of 630) tested positive for hrHPV infections (Table 2). During 9,512 PMO from 2012 through May 2015, we documented 95 women with cleared HPV infections (Table 2), of whom 62 had cleared lrHPV events, 51 had cleared hrHPV events, and 18 had cleared lrHPV and hrHPV events. In age-adjusted analysis, we found that married women were more likely to clear lrHPV infections than were unmarried women (Table 3).

**Table 1:**
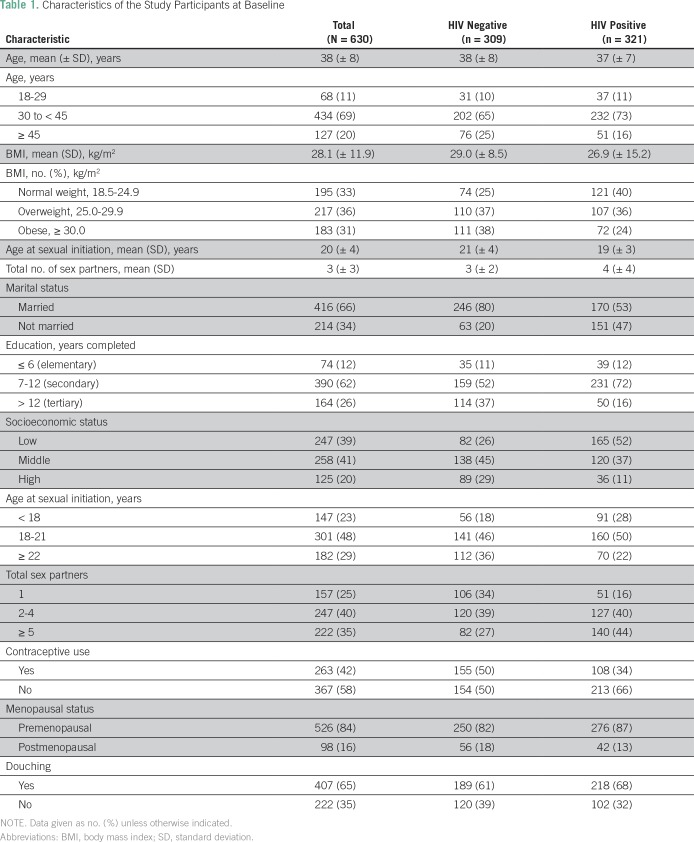
Characteristics of the Study Participants at Baseline

**Table 2:**
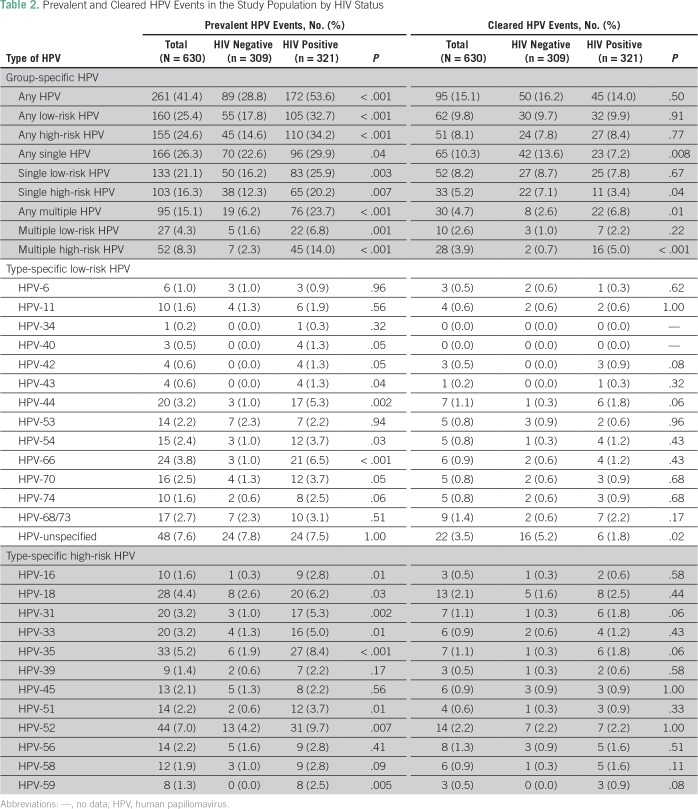
Prevalent and Cleared HPV Events in the Study Population by HIV Status

**Table 3:**
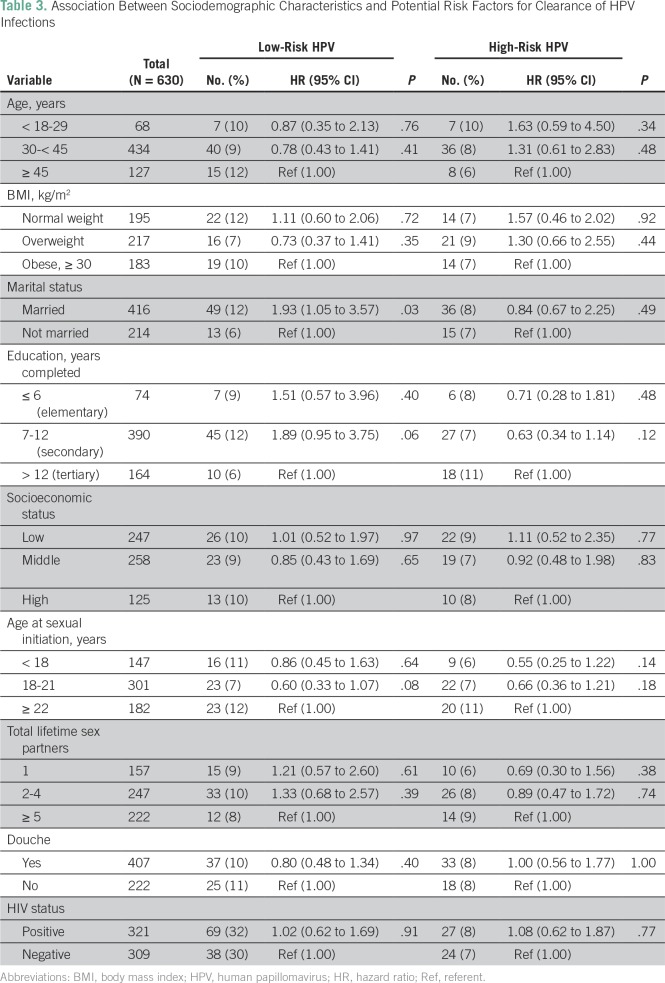
Association Between Sociodemographic Characteristics and Potential Risk Factors for Clearance of HPV Infections

### HIV-Negative Women

HIV-negative women contributed 6,113 PMO of follow-up. Any type of HPV infection of the cervix occurred in 29% (89 of 309) of the HIV-negative women at baseline. Some 16% (50 of 309) of the women cleared infections by any HPV during follow-up, giving a clearance rate (CR) of 25.3/1,000 PMO (Table 4). The cleared infections involved lrHPV types in 10% (30 of 309; CR, 9.21/1,000 PMO) and hrHPV types in 8% (24 of 309; CR, 8.83/1,000 PMO) of the women. The clearance rates of the lrHPVs and hrHPVs were not statistically significantly different (*P* = .37).

**Table 4:**
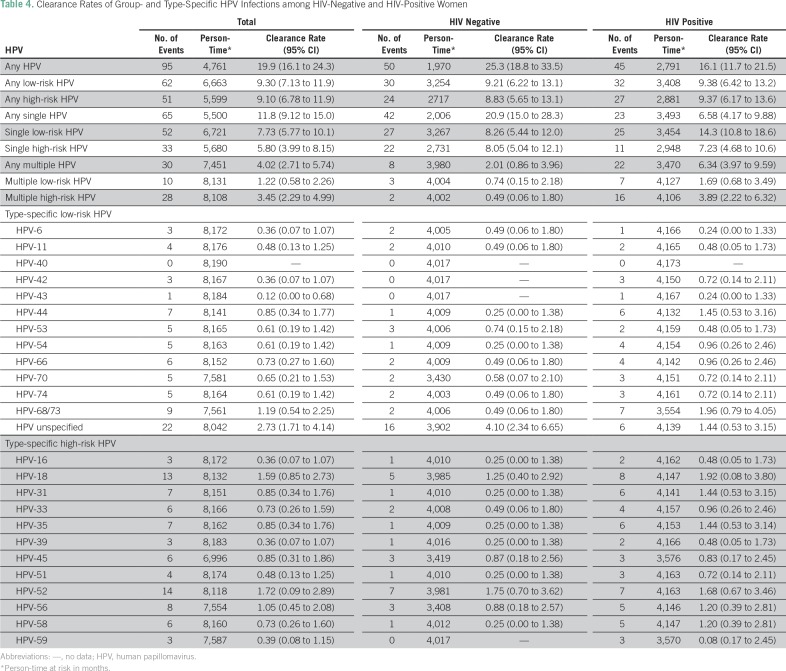
Clearance Rates of Group- and Type-Specific HPV Infections among HIV-Negative and HIV-Positive Women

The most common lrHPV types present at baseline were unspecified HPV (8%, 24 of 309), HPV 68/73 (2.3%, seven of 309), and HPV 53 (2.3%, seven of 309). The infections most likely to be cleared during follow-up were those caused by unspecified HPV types (5%, 16 of 309; CR, 4.10/1,000 PMO) and HPV-53 (0.9%, three of 309; CR, 0.74/1,000 PMO).

HPV-52 (4%, 13 of 309) and HPV-18 (2.6%, eight of 309) were the most common hrHPV types present at baseline. These HPV types were also the most likely to be cleared, with HPV-52 cleared in 2.2% (seven of 309; CR, 1.75/1,000 PMO) of the women and HPV-18 cleared in 1.6% of the women (five of 309; CR, 1.25/1,000 PMO).

Single cervical infection by any type of HPV occurred in 22.6% (70 of 309) of the HIV-negative women at baseline. This was cleared in 13.6% (42 of 309; CR, 20.9/1,000 PMO) of the women during follow-up. The cleared infections involved lrHPV types in 8.7% (27 of 309; CR, 8.26/1,000 PMO) and hrHPV types in 7.1% (22 of 309; CR, 8.05/1,000 PMO). The clearance rates of single lrHPV and hrHPV were not statistically significantly different (*P* = .44).

Multiple HPV infections of the cervix were detected in 5.8% (18 of 309) of the HIV-negative women at baseline. These infections were cleared in 2.6% (eight of 309; CR, 2.01/1,000 PMO) of the women during follow-up. The cleared infections were lrHPV types in 1.0% (three of 309; CR, 0.74/1,000 PMO) and hrHPV types in 0.7% (two of 309; CR, 0.49/1,000 PMO). The clearance rates of multiple lrHPV and hrHPV were not statistically significantly different (*P* = .65).

### HIV-Positive Women

HIV-positive women contributed 3,399 PMO of follow-up. Any type of HPV infection of the cervix was detected in 53.6% (172 of 321) of these women at baseline. Some 14.0% (45 of 321) of the women cleared the infections by any HPV during follow-up, yielding a CR of 16.1/1,000 PMO. The cleared infections involved lrHPV types in 9.9% (32 of 321; CR, 9.38/1,000 PMO) and hrHPV types in 8.4% (27 of 321; CR, 9.37/1,000 PMO). Statistically, the clearance rates of any lrHPV and hrHPV were not significantly different (*P* = .37).

The most common lrHPV types present at baseline were unspecified HPV (7.5%, 24 of 321) and HPV-66 (6.5%, 21 of 321). The women were most likely to clear infections by HPV-68/73 (2.2%, seven of 321; CR, 1.96/1,000 PMO), HPV-66 (1.8%, six of 321; CR, 1.45/1,000 PMO), and unspecified HPV (1.8%, six of 321; CR, 1.44/1,000 PMO). The most common hrHPV types present at baseline were HPV-52 (9.7%, 31 of 321) and HPV-35 (8.4%, 27 of 321). Infections with HPV-18 (2.5%, eight of 321; CR, 1.92/1,000 PMO) and HPV-52 (2.2%, seven of 321; CR, 1.68/1,000 PMO) were most likely to be cleared.

Single HPV infections of the cervix were detected in 29.9% (96 of 321) of the HIV-positive women at baseline. Some 7.2% (23 of 321) of the women had cleared these infections during follow-up, giving a CR of 6.58/1,000 PMO. The cleared infections involved lrHPV types in 7.8% (25 of 321; CR, 14.3/1,000 PMO) and hrHPV types in 3.4% (11 of 321; CR, 7.23/1,000 PMO). The clearance rate for single lrHPV infections was significantly higher than that of single hrHPV (*P* = .01).

Multiple cervical HPV infections were detected in 23.7% (76 of 321) of the women at baseline. These infections were cleared by 6.8% (22 of 321; CR, 6.34/1,000 PMO) of the women during follow-up. Infection by lrHPV types (2.2%, seven of 321; CR 1.69/1,000 PMO) were less likely to be cleared than infection by hrHPV types (5.0%, six of 321; CR, 3.89/1,000 PMO) in these women (*P* = .01).

### Impact of HIV Infection

**Fig 1:**
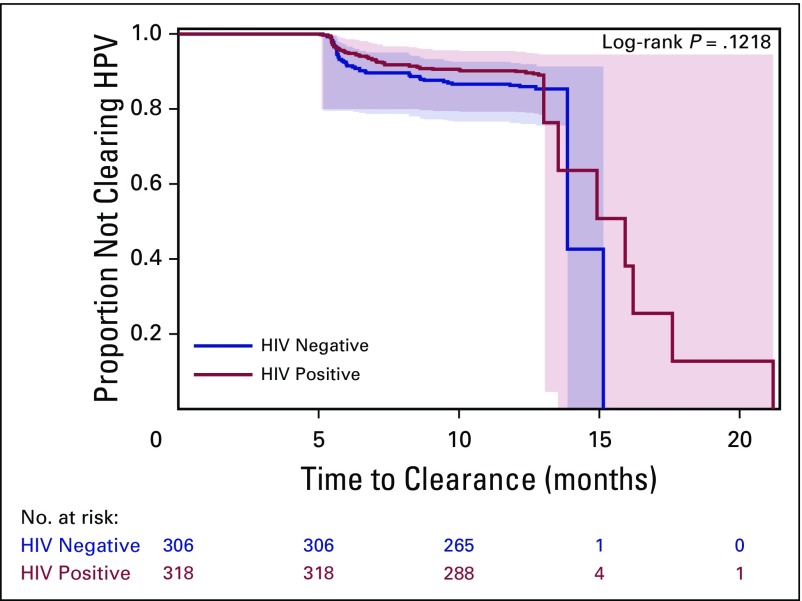
Kaplan-Meier curves for clearance of any HPV by HIV status. Bands in the graph are Hall-Wellner 95% confidence bands. HPV, human papillomavirus.

The distribution of low-risk and high-risk, cleared, any HPV, single HPV, and multiple HPV by HIV status is shown in Table 2. At baseline, HIV-positive women were more likely to be infected with any HPV (*P* < .001), any lrHPV (*P* < .001), any hrHPV (*P* < .001), any single HPV (*P* = .04), any single lrHPV (*P* = .003), any single hrHPV (*P* = .007), any multiple HPV (*P* = .001), multiple lrHPV (*P* = .001), and multiple hrHPV (*P* ≤ .001) compared with HIV-negative women. Figure 1 provides Kaplan-Meier curves illustrating the proportion of women who cleared infections with any HPV type during the follow-up period, by HIV status. As seen in the figure, there was no significant difference between clearance of any HPV by HIV status (log-rank test *P* = .12). In multivariate analyses adjusted for age and marital status, the HRs for cleared infections in HIV-positive women compared with HIV-negative women were 0.95 (95% CI, 0.54 to 1.65; *P* = .85) for any hrHPV; 2.12 (95% CI, 1.02 to 4.40; *P* = .04) for single hrHPV, and 0.13 (95% CI, 0.03 to 0.58; *P* = .007) for multiple hrHPV (Table 5).

**Table 5:**
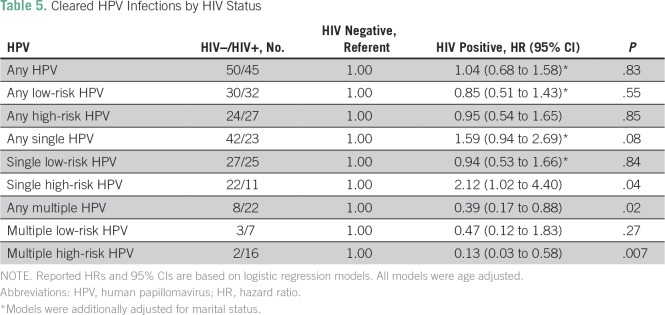
Cleared HPV Infections by HIV Status

## DISCUSSION

Our study provides a comprehensive description of cleared lrHPV and hrHPV infections in a cohort of HIV-negative and HIV-positive women in Nigeria, West Africa. We observed that, in general, the rate and risk of cleared lrHPV and hrHPV infections were not substantially modified by HIV status. However, HIV-positive women had 83% significant reduction in their likelihood to clear multiple hrHPV infections, compared with HIV-negative women.

We did not find significant associations between sociodemographic factors, lifestyle or sexual behavior, and lrHPV or hrHPV clearance. This is similar to the results of a study of Inuit women in Canada.^13^ However, we observed that the HR for any lrHPV clearance was 93% higher among married women in our study compared with unmarried women. This may be a reflection of lower sexual networking among married women and greater propensity of lrHPV to be cleared compared with hrHPV. Another Canadian study showed that the odds ratio of clearing any HPV was 29% higher among women who were married or living with a partner compared with those who were not; however, the results were not statistically significant.^13^

Among the HIV-negative women in our study population aged 18 to 61 years, 15% cleared their baseline HPV infections at a median follow-up time of 9 months. Another study found that 52% of the participants aged 15 to 49 years cleared their baseline HPV infections at a median follow-up time of 13.5 months,^14^ whereas 36% of participants aged 15 to ≥ 60 years cleared their incident HPV infections at a median follow-up time of 36 months.^15^ The CR of any HPV among HIV-positive women in our study population was high (16.1/1,000 PMO). In a prospective study of 215 HIV-positive Indian women, the CR for any HPV was 18.3/100 person-years (0.15/1,000 PMO).^16^ In general, our estimates of HPV clearance may differ from that of other studies because of differences in the study design and procedures, the age range of the participants, differences in lifestyle in different geographic regions, duration of follow-up, and method of HPV detection.

Although we did not observe any association between HIV seropositivity and clearance of any HPV and single HPV, HIV-positive women in our study had a significant reduction in the likelihood of clearing any multiple HPV (adjusted HR, 0.39; 95% CI, 0.17 to 0.88) and multiple hrHPV (adjusted HR, 0.13; 95% CI, 0.03 to 0.58), compared with HIV-negative women. Although some studies documented that HIV-positive women have lower likelihood of clearing cervical HPV infections, compared with HIV-negative women,^9,17^ another study found the effect estimates for HPV clearance were not substantially modified by HIV serostatus.^18^

Although we and others found that HIV infection may be an important risk factor of HPV clearance, the mechanism of its action in HPV-induced carcinogenesis has not been well elucidated. It was reported that HIV-related immunodeficiency may alter the relative carcinogenicity of hrHPV types.^19^ HPV-16 infections may be less affected by immunodeficiency than other hrHPV types^20,21^ and it tends to be underrepresented compared with other hrHPV types in HIV-positive women.^19,22^ In our study, HPV-16 was the seventh most common prevalent type among HIV-positive women, after HPV-52, -35, -18, -31, -33, and -51. However, we were unable to examine the relationship between hrHPV types and changes in immunodeficiency, because we did not have data on clinical correlates of HIV infection, such as CD4 cell count.

Our study has several strengths. We collected detailed data on numerous exposures using validated procedures and we were able to prospectively follow-up a large number of participants, which allowed us to examine the impact of HIV on the clearance of lrHPV and hrHPV. Although we did not have HIV clinical information such as duration of HIV infection, use of antiretroviral therapy, viral load, or CD4 cell count that may be associated with clearance of HPV infection among HIV-positive women, we excluded from our analyses HIV-positive women who were severely ill. However, in another study in which clinical features of HIV were examined in relation to HPV, no association was found.^23^

We conducted detailed analyses of the clearance rates of infections with specific types and groups of lrHPV and hrHPV in HIV-positive and HIV-negative women. We described the impact of HIV on the risk of HPV clearance among women in West Africa. HIV infection was significantly associated with reduced likelihood of clearing multiple hrHPV infections.
